# Water Supplementation Reduces Copeptin and Plasma Glucose in Adults With High Copeptin: The H_2_O Metabolism Pilot Study

**DOI:** 10.1210/jc.2018-02195

**Published:** 2018-12-18

**Authors:** Sofia Enhörning, Louise Brunkwall, Irina Tasevska, Ulrika Ericson, Jenny Persson Tholin, Margaretha Persson, Guillaume Lemetais, Tiphaine Vanhaecke, Alberto Dolci, Erica T Perrier, Olle Melander

**Affiliations:** 1 Department of Clinical Science, Lund University, Skåne University Hospital, Malmö, Sweden; 2 Department of Endocrinology, Skåne University Hospital, Malmö, Sweden; 3 Department of Internal Medicine, Skåne University Hospital, Malmö, Sweden; 4 Hydration and Health Department, Danone Research, Palaiseau, France

## Abstract

**Objective:**

Because elevated copeptin, a marker of vasopressin, is linked to low water intake and high diabetes risk, we tested the effect of water supplementation on copeptin and fasting glucose.

**Design, Setting, and Participants:**

Thirty-one healthy adults with high copeptin (>10.7 pmol · L^−1^ in men and >6.1 pmol·L^−1^ in women) identified in a population-based survey from 2013 to 2015 and with a current 24-hour urine osmolality of >600 mOsm · kg^−1^ were included.

**Intervention:**

Addition of 1.5 L water daily on top of habitual fluid intake for 6 weeks.

**Main outcome measure:**

Pre- and postintervention fasting plasma copeptin concentrations.

**Results:**

Reported mean water intake increased from 0.43 to 1.35 L · d^−1^ (*P* < 0.001), with no other observed changes in diet. Median (interquartile range) urine osmolality was reduced from 879 (705, 996) to 384 (319, 502) mOsm · kg^−1^ (*P* < 0.001); urine volume increased from 1.06 (0.90, 1.20) to 2.27 (1.52, 2.67) L · d^−1^ (*P* < 0.001); and baseline copeptin decreased from 12.9 (7.4, 21.9) pmol · L^−1^ to 7.8 (4.6;11.3) pmol · L^−1^ (*P* < 0.001). Water supplementation reduced fasting plasma glucose from a mean (SD) of 5.94 (0.44) to 5.74 (0.51) (*P* = 0.04). The water-associated reduction of both fasting copeptin and glucose concentration in plasma was most pronounced in participants in the top tertile of baseline copeptin.

**Conclusions:**

Water supplementation in persons with habitually low water consumption and high copeptin levels is effective in lowering copeptin. It appears a safe and promising intervention with the potential of lowering fasting plasma glucose and thus reducing diabetes risk. Further investigations are warranted to support these findings.

## Teaser

In individuals with high plasma concentration of the vasopressin marker copeptin, increased water intake of 1.5 L daily during 6 weeks significantly reduced copeptin and fasting glucose.

Type 2 diabetes is a growing health problem worldwide that substantially increases the risk for cardiovascular disease and death. In fact, cardiovascular risk is increased even at nondiabetic levels of glycemia (1, 2), highlighting the importance of actions that lower glucose levels and thus prevent diabetes in broad segments of the population.

It is suggested that vasopressin, a small, short-lived peptide released from the posterior pituitary gland, may be a key player in the development of metabolic and cardiovascular disease. Copeptin, the stable C-terminal cleavage product of the vasopressin precursor is identified as an independent risk factor for diabetes and cardiovascular disease. Copeptin is released in a 1:1 ratio with vasopressin and is a reliable measure of vasopressin concentration (3–5). We previously showed that the 25% of the population with the highest plasma concentration of copeptin [>6.1 · L^−1^ in women and >10.7 · L^−1^ in men in a large Swedish population cohort (6)] have a markedly increased risk for new-onset type 2 diabetes, a finding later replicated in other large prospective population-based studies (7, 8). Furthermore, participants with high copeptin concentration have an increased risk for all components of the metabolic syndrome (6, 9, 10), as well as cardiovascular disease and premature mortality; this increased risk is seen both in patients with diabetes and in the general population (11–13). It is not yet known whether the link between high levels of circulating vasopressin (copeptin) and metabolic disturbances is causal; however, both manipulation of vasopressin concentration by altered water intake in rodents (14) and a human Mendelian randomization study (15) have suggested a causal relationship.

One of vasopressin's many physiological functions is to maintain constant plasma osmolality in conditions of low water intake by mediating water reabsorption through vasopressin 2 receptors in the renal collecting ducts (16). Furthermore, there are many potential ways in which vasopressin could influence glucose metabolism; in particular, the vasopressin 1a receptor, which is widely expressed in the body, is involved in glycogenolysis and gluconeogenesis in the liver (17, 18). Additionally, the vasopressin 1b receptor is expressed in the anterior pituitary gland, where it, together with corticotrophin-releasing hormone, mediates release of adrenocorticotrophin hormone and thus is important for the endocrine stress response (19, 20). Vasopressin is also reported to be locally released within the adrenal gland, where it is suggested to mediate cortisol release (21). In addition, vasopressin 1b receptor is expressed in the pancreas, where it mediates glucagon and insulin secretion (22, 23).

Individuals with habitually low water intake have higher vasopressin concentrations than individuals with habitually higher water intake (24, 25), whereas increased water intake over days to weeks effectively lowers circulating vasopressin (or copeptin) (25, 26). Epidemiological evidence has shown that high water intake is associated with better glucose control (27), and rats with water-induced reduction in circulating vasopressin show increased insulin sensitivity (14). To further investigate the role of a reduction of vasopressin on glucose metabolism and glucoregulatory hormones, we recently conducted a short-term water intervention experiment in 37 healthy humans; in those participants, we found that copeptin could be effectively lowered by increasing water intake both acutely (by a rapid oral water load of 1 L) and after 1 week of 3 L of extra water per day (28). One third of the participants with the greatest copeptin reduction after the water week (water responders) were the ones with habitually high urine osmolality, low urine volume, and high baseline copeptin (*i.e.,* signs of low water intake). Among these water responders, we also found a significant water-induced reduction of fasting glucagon concentration.

We hypothesize that higher vasopressin levels, as a result of low water intake, stimulates vasopressin 1a receptor and vasopressin 1b receptor and may result in higher plasma glucose levels, which may be reversed by water-induced reduction of vasopressin. Additionally, a proportion of participants with elevated copeptin (high vasopressin) have high diabetes risk (6–8) and indices of low water intake and respond with copeptin reduction upon water supplementation (28). Therefore, this segment of the population is a suitable target for a hydration intervention. We recently initiated such a study in which 12 months of hydration (1.5 L water daily on top of habitual fluid intake) vs control therapy (no change in water intake) will be tested, with difference in pre-compared with postintervention change in fasting plasma glucose as the primary outcome (NCT03422848).

Here we present the results from a pilot study that tested whether 6 weeks of water supplementation (1.5 L additional water per day) in persons with low water intake and high copeptin levels could significantly alter hydration markers in general and reduce plasma copeptin in particular. Furthermore, because fasting plasma glucose is the primary endpoint in the long-term randomized trial, we also explored whether this 6-week water intervention could significantly reduce fasting plasma glucose concentrations.

## Materials and Methods

### Recruitment and inclusion process

**Figure 1.**
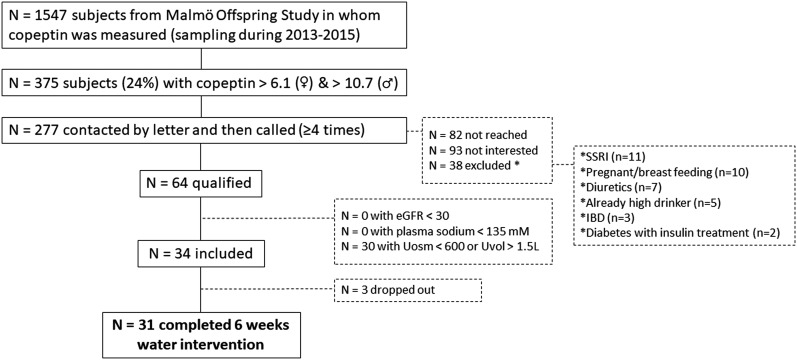
Recruitment and inclusion process. IBD, inflammatory bowel disease.

All participants were selected from an ongoing population study in the Scania region of southern Sweden, the Malmö Offspring Study (MOS) (http://www.med.lu.se/mos) (29). Selection was performed by analyzing copeptin concentrations from plasma samples, frozen at −80°C, that had been obtained from this cohort from 2013 to 2015. Individuals aged 20 to 75 years and had high plasma copeptin concentrations [>6.1 pmol · L^−1^ in women and >10.7 pmol · L^−1^ in men, corresponding to the top quartile of copeptin in the Malmö Diet and Cancer–Cardiovascular Cohort (6)] were deemed eligible for the current study and contacted by letter and telephone (Fig. 1). Individuals interested in participation who had no medication or disease necessitating immediate exclusion (see exclusion criteria below) provided informed consent and underwent a second eligibility check (including providing blood samples for measurement of plasma sodium and creatinine and 24-hour urine for measurement of urine volume and osmolality) before definitive enrollment. In addition to high copeptin and age range as specified above, inclusion criteria were as follows: 24-hour urine osmolality ≥ 600 mOsm · kg^−1^ water and 24-hour urine volume ≤ 1.5 L (Fig. 1) (*i.e.,* laboratory findings that, together with high copeptin, indicated low water intake). Exclusion criteria were as follows: pregnancy or breastfeeding; plasma sodium < 135 mM; use of diuretics, lithium, or selective serotonin reuptake inhibitors; chronic kidney disease (estimated glomerular filtration rate (eGFR) < 30 mL/min per 1.73 m^2^); heart failure; type 1 diabetes or type 2 diabetes treated with insulin; inflammatory bowel disease; and vulnerable persons (those with a legal guardian or loss of personal liberty) (Fig. 1). Thus, individuals with increased risk for hyponatremia (*i.e.,* use of diuretics, lithium, or selective serotonin reuptake inhibitors; heart failure) were not eligible to participate for safety reasons.

The study was approved by the ethics committee of Lund University and performed in accordance with the ethical standards laid down in the 1964 Declaration of Helsinki and its later amendments. All participants provided written informed consent before their inclusion in the study.

### Study visits

Clinic visits were performed at baseline, after 3 weeks of the intervention, and after 6 weeks (*i.e.,* at the end of the intervention), and were scheduled from Monday to Friday to avoid changes in dietary habits due to different routine during the weekend. At baseline and at the 6-week visit, participants arrived at the clinic between 7:45 am and 9:15 am in a fasting state. At the 3-week visit, the participants arrived at the clinic at any time during the day.

### Intervention

**Figure 2.**
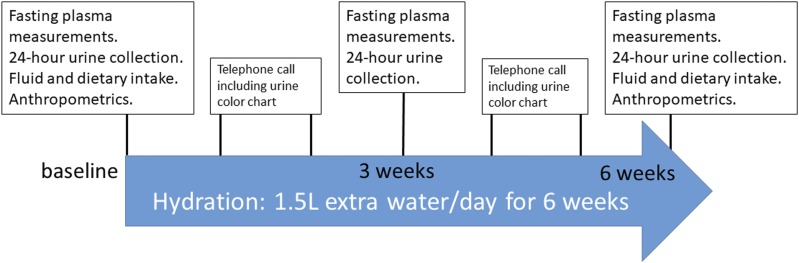
Study protocol.

Participants were instructed to add 1.5 L of plain water daily on the top of habitual intake (intervention) for 6 weeks (Fig. 2). Adherence to high water intake was achieved by coaching at the clinic visits and by regular telephone contacts with the participants (Fig. 2). Participants were provided with a paper urine color scale (30) to use at home to facilitate and optimize adherence to the water intervention.

### Protocol

At all study visits, plasma creatinine, sodium, potassium, urea, and osmolality were analyzed. At baseline and at the 6-week visit, additional measurements of plasma copeptin, C-reactive protein, and fasting glucose were performed, and anthropometry was assessed.

Participants collected 24-hour urine samples the day before each study visit. Upon awakening, participants voided and discarded this first morning urine sample. All subsequent urine produced throughout the day and overnight was collected in a single container. The next morning, upon awakening, participants produced a final first morning void, which completed the 24-hour collection. Participants delivered the complete, fresh 24-hour urine samples to the clinic during their study visit that day. We used 24-hour urine volume and osmolality, together with home reminders using the urine color scale, to motivate the participants to keep up or, in case of indications of lack of adherence, increase their water intake.

Before the baseline and the 6-week visit, most participants (n = 24) recorded usual food and fluid intake by using Riksmaten 2010, a validated web-based 4-day record tool developed by the Swedish National Food Administration and used in the latest national diet survey in Swedish adults (31). Blood pressure following a 5-minute rest, current medication, and medical history were also recorded.

### Laboratory measurements

Fasting plasma copeptin concentration was measured by using a KRYPTOR Compact Plus device and commercially available chemiluminescence sandwich immunoassay copeptin ProAVP kit with coated tubes from samples stored at −80°C (BRAHMS Copeptin proAVP KRYPTOR; ThermoFisher Scientific). All other plasma laboratory analyses were performed at the certified University Hospital’s central clinical laboratory. This included measurement of glucose (COBAS, Roche Diagnostics, Rotkreuz, Switzerland), creatinine, sodium, potassium, urea, osmolality, hematocrit, and C-reactive protein. Urine osmolality was measured in 24-hour urine samples at the clinical site by using an i-Osmometer basic (Löser, Speyer, Germany). The 24-hour urine collections followed procedures developed at the Department of Endocrinology, Skåne University Hospital Malmö, Sweden, and consisted of a comprehensible written instruction aimed at ensuring accurate and complete collection of urine.

### Statistics

Significance of differences between baseline and postintervention measurements was tested by using a paired *t* test or Wilcoxon paired-rank test, depending on distribution. Furthermore, participants were divided into subgroups according to baseline copeptin concentration (top tertile of copeptin *vs* tertiles 1 and 2). Significance of differences between these two subgroups was tested by using an independent sample *t* test or Mann-Whitney *U* test depending on normality; a Fisher exact test was used for dichotomous variable. Finally, significant difference in change of fasting glucose was tested in subgroups defined by baseline copeptin using a one sample *t* test. SPSS statistical software version 24 (IBM Inc., Chicago, IL) was used for all analyses. A two-sided *P* value < 0.05 was considered to indicate a statistically significant difference.

## Results

### Recruitment and dropout rate

Of the 1547 MOS participants in whom copeptin was measured, 375 (24%) had high copeptin concentrations (>6.1 pmol · L^−1^ in women and >10.7 pmol · L^−1^ in men). Of these, 277 were contacted, and after the recruitment process, 34 individuals were included in the study (Fig. 1). The dropout rate during the intervention was 9% (n = 3). In two cases, the dropout was due to infections without any suspicion of link to the ongoing water intervention. In one case the dropout was for personal reasons. Among the 31 individuals that completed the 6-week water intervention, the mean age was 43 (range, 22 to 66) years; 61% of participants were men. The median (25th, 75th percentiles) copeptin concentration in the frozen plasma samples collected in the MOS cohort from 2013 to 2015 was 11.8 (10.1, 13.8). None of the included study participants had a diabetes diagnosis, diabetes medication, or elevated baseline fasting blood glucose (≥7 mmol/L).

The recruitment and inclusion process was rather time-consuming; 8.1 individuals had to be called (up to four times) for one inclusion. The dropout rate was <9%, which we considered to be low given that the study included healthy volunteers. We expect a higher dropout rate in the long-term (1 year) randomized trial, primarily due to the longer duration of the study.

### Baseline visit

The median copeptin concentration at the pilot baseline (*i.e.,* 2.8 ± 0.8 years after population screening) was similar to the median concentration obtained in the MOS samples but with a wider spread (Table 1). In the baseline samples, copeptin concentration remained above the screening thresholds (>6.1 pmol · L^−1^ in women and >10.7 pmol · L^−1^ in men) in 61% of the individuals. Urine volume and osmolality at baseline are shown in Table 1.

**Table 1.** Plasma Copeptin, Urine Osmolality, and Urine Volume: Baseline and During and After Water Intervention

Variable	Baseline	After 3 Wk	After 6 Wk	*P* Value (Wilcoxon Paired Rank Test)
Plasma copeptin, pmol · L^−1^	12.9 (7.4, 21.9)	NA	7.8 (4.6, 11.3)	<0.001^*a*^
Urine osmolality, mOsm · kg^−1^ H_2_O	879 (705, 996)	374 (327, 514)	384 (319, 502)	<0.001^*a*^
				<0.001^*b*^
Urine volume, L · 24 h^−1^	1.06 (0.90, 1.20)	2.37 (1.87, 2.61)	2.27 (1.52, 2.67)	<0.001^*a*^
				<0.001^*b*^

Data given as median (25th,75th percentiles).

Abbreviation: NA, not applicable.

^a^ Six weeks *vs* baseline.

^b^ Three weeks *vs* baseline.

### Effects from increased water intake

Increased water intake resulted in improved hydration, as represented by significantly lower 24-hour urine osmolality and significantly higher 24-hour urine volume at both the 3-week and 6-week measurement after the water intervention (Table 1). The 4-day web-based diet and fluid record showed significantly increased intake of drinking water (carbonated and noncarbonated), with no significant changes in other beverages or in water from food moisture and no significant changes in other dietary variables (Table 2).

**Table 2.** Results of 4-Day Web-Based Diet and Fluid Assessment at Baseline and After 6 Weeks of Water Intervention

Variable	Baseline	After 6 Wk	*P* Value (Paired *t* Test)
Energy, kcal · day^−1^	2020 (1817–2222)	1955 (1715–2196)	0.39
Carbohydrates, g · day^−1^	204 (180–229)	201 (170–233)	0.77
Fat, g · day^−1^	82 (74–92)	77 (68–86)	0.19
Protein, g · day^−1^	75 (67–83)	78 (69–86)	0.32
Fiber, g · day^−1^	17 (7–19)	16 (14–18)	0.51
Fruit and berries, g · day^−1^	49 (23–75)	62 (31–93)	0.17
Vegetables, g · day^−1^	125 (99–150)	123 (93–154)	0.94
Meat, g · day^−1^	97 (71–122)	89 (73–106)	0.48
Alcohol, g · day^−1^	17 (7–28)	16 (5–26)	0.63
Coffee, g · day^−1^	217 (123–312)	196 (86–304)	0.41
Tea, g · day^−1^	61 (28–95)	59 (16–101)	0.88
Drinking water, L · day^−1^	0.43 (0.27–0.58)	1.35 (1.00–1.71)	<0.001
Total water, L · day^−1^^*a*^	1.84 (1.60–2.07)	2.69 (2.28–3.11)	<0.001

n = 24. Data given as mean (95% CI).

^a^ Water from food moisture and fluids.

The water intervention resulted in a significant median (25th, 75th percentiles) copeptin reduction of 4.2 (0.8, 9.4) pmol · L^−1^ compared with baseline (Table 1). Individuals with the highest baseline copeptin concentrations (copeptin tertile 3) obtained a more pronounced water-induced reduction of copeptin than did individuals with moderately elevated baseline copeptin concentrations (copeptin tertiles 1 and 2) but were otherwise similar in clinical characteristics (Table 3).

**Table 3.** Comparison of Baseline Plasma Parameters and Water-Induced Copeptin Reduction Between Individuals With the Highest Baseline Copeptin Concentrations (Copeptin Tertile 3) and Those in Copeptin Tertiles 1 and 2

Variable	Copeptin Tertile 3 ^*a*^ (n = 10)	Copeptin Tertiles 1 and 2 ^*a*^ (n = 21)	*P* Value ^*b*^
Baseline Copeptin, pmol · L^−1^^*c*^	35.2 (21.0–66.5)	8.7 (5.6–13.1)	<0.001^*d*^
*△*Copeptin, pmol · L^−1^^*c*^^,^^*e*^	28.2 (8.1–58.6)	1.6 (–0.3–4.5)	<0.001^*d*^
Cardiometabolic drugs, n (%)^*f*^	1 (10)	5 (24)	0.35^*g*^
Body mass index, kg · m^−2^	26.7 (5.2)	27.7 (6.3)	0.63
Plasma osmolality, mOsm · kg^−1^	297.6 (2.6)	295.3 (4.5)	0.09
Plasma sodium, mmol · L^−1^	141.7 (1.3)	141.0 (1.8)	0.24
C-reactive protein, mg · L^−1^^*c*^	0.9 (0.7–4.5)	2.4 (1.2–5.9)	0.28^*d*^
Age, y	38.0 (15.3)	45.8 (13.3)	0.19

Unless otherwise noted, data are expressed as mean (SD).

^a^ Pooled sex-specific tertiles.

^b^ Independent sample *t* test if not otherwise specified.

^c^ Expressed as median (interquartile range).

^d^ Mann-Whitney *U* test.

^e^ Difference between baseline and 6-wk copeptin concentrations.

^f^ Aspirin, statins, or antihypertension medications.

^g^ Fisher exact test.

**Figure 3.**
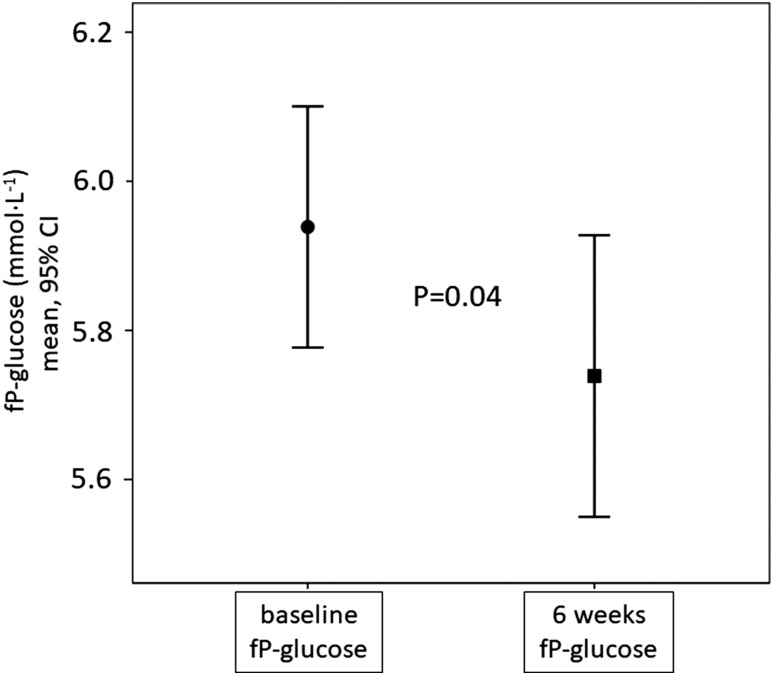
Fasting glucose concentrations at baseline and after 6 wk of water intervention. fP, fasting plasma.

**Figure 4.**
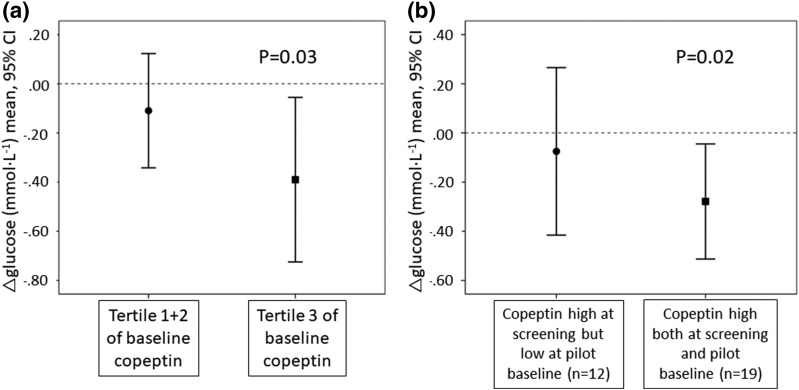
Changes in fasting glucose (difference between 6-wk and baseline glucose concentrations). (a) Participants with high baseline copeptin values (tertile 3) vs participants with lower baseline copeptin values (tertiles 1 and 2). (b) Participants with high copeptin (>6.1 pmol · L^−1^ in women and >10.7 pmol · L^−1^ in men) both at screening and at pilot vs participants with high copeptin at screening but low copeptin at pilot baseline.

Water supplementation significantly reduced mean (SD) fasting plasma glucose from 5.94 (0.44) to 5.74 (0.51) mmol · L^−1^ (*P* = 0.04) (Fig. 3), an effect that was driven by participants within the top tertile of baseline copeptin [Fig. 4(a)] and by the 61% of participants whose copeptin concentration remained high (>6.1 pmol · L^−1^ in women and >10.7 pmol · L^−1^ in men) from population screening to pilot baseline investigation ∼3 years later [Fig. 4(b)].

When blood parameters, eGFR, and anthropometric measures were compared between baseline and 6 weeks, we found no change in potassium, osmolality, erythrocyte volume fraction, eGFR, body weight, and systolic or diastolic blood pressure (Table 4). Plasma sodium was slightly but significantly reduced after the 6-week water intervention (*P* = 0.001); however, no participant developed hyponatremia. Plasma urea was not significantly affected by the intervention (*P* = 0.055) (Table 2).

**Table 4.** Plasma Parameters and Anthropometric Values at Baseline and After 6 Weeks of Water Intervention

Variable	Baseline	After 6 Wk	*P* Value (Paired *t* Test)
fP sodium, mmol · L^−1^	141.2 (1.65), 137–145	140.1 (1.41), 137–144	0.001
fP potassium, mmol · L^−1^	3.76 (0.22), 3.2–4.1	3.78 (0.20), 3.4–4.2	0.82
fP osmolality, mOsm/kg H_2_O	296 (4.1), 288–306	295 (3.4), 288–300	0.23
fP urea, mmol · L^−1^	4.61 (1.08), 2.7–7.5	4.26 (1.16), 2–7	0.055
*S*-erythrocyte volume fraction	0.42 (0.03), 0.38–0.46	0.41 (0.03), 0.3–0.5	0.18
GFR (MDRD), mL · min^−1^·1.73 m^−2^	90.9 (15.7), 63.4–116.3	92.8 (15.7), 60.9–126.2	0.10
Weight, kg	84.8 (20.5), 44.9–136.0	84.5 (20.0), 44.9–136.4	0.43
Systolic blood pressure, mm Hg	119 (14), 91–144	121 (14), 89–148	0.32
Diastolic blood pressure, mm Hg	81.3 (10.1), 58–98	79.3 (9.6), 60–100	0.15

Data are given as mean (SD), range.

Abbreviations: fP, fasting plasma; GFR, glomerular filtration rate; MDRD, Modification of Diet in Renal Disease.

## Discussion

The key finding of this study was that 1.5 L water supplementation per day during 6 weeks in persons with low water intake and high plasma copeptin significantly reduced plasma copeptin concentrations, reduced urine osmolality, and increased urine volume. Surprisingly, despite the rather short intervention period, a small but statistically significant reduction of fasting plasma glucose was observed, which could not be explained by any change in weight or dietary intake except from increased water intake. Additionally, the reductions in both copeptin and glucose were driven by the participants with the highest copeptin concentrations at baseline, suggesting that water supplementation may be particularly effective in individuals with high plasma copeptin and thus increased cardiometabolic risk.

We previously showed that individuals who obtained the greatest reduction in copeptin from increased water intake (water responders) are characterized by high copeptin, high urine osmolality, and low urine volume [*i.e.,* indices of low water intake and insufficient hydration (28)], whereas individuals who already drink moderate to high amounts of water respond with a modest copeptin reduction from increased hydration (26, 28). These previous data underlined the importance of targeting individuals with indices of low water intake in the current study and resulted in using high copeptin, high urine osmolality, and low urine volume as inclusion criteria. The study was designed to investigate the copeptin-lowering effect from increased water intake in persons with low water intake, as well as to test study logistics, safety, adherence, and dropout rate before the start of a long-term randomized trial. We chose a daily water supplementation of 1.5 L because this volume was sufficient to show excellent separation of hydration markers between intervention and control groups in a previous randomized trial examining the effects of increased water intake on kidney function in patients with chronic kidney disease (32), while still remaining a manageable goal for daily water increase.

The achieved median difference in 24-hour urine volume between the baseline and 6-week measurement was around 1.2 L, and the mean difference in self-reported daily drinking water was around 0.9 L, suggesting that when an additional 1.5 L of water is prescribed, the actual increase achieved by participants will be slightly less than the targeted added volume. We observed the same discrepancy in our previous water intervention experiment (28).

Vasopressin is established as an independent risk factor for diabetes, the metabolic syndrome, chronic kidney disease, cardiovascular disease, and premature death in the population (6, 10–12, 33, 34). In a population-based Swedish cohort study, we previously showed that the risk for new-onset diabetes development after a mean follow-up of 12.6 years was three- to fourfold in the 25% of the population with the highest copeptin concentrations than in the 25% with the lowest concentrations, after adjustment for other diabetes risk factors (6). Thus, individuals with habitually high copeptin concentrations and other signs of low water intake not only have the most prominent copeptin-lowering effect from water but also exhibit a considerably greater diabetes risk; thus, this segment of the population is a target population for water interventions. To represent this segment of the population, the copeptin cutoff level used as an inclusion criterion in this study corresponded to the top 25% of the previous population-based study (*i.e.,* >6.1 · L^−1^ in women and >10.7 · L^−1^ in men). Although there is no established reference range for normal *vs* "at-risk" copeptin concentration in the general population, similar copeptin concentrations were reported in the top 25% of a Dutch population-based cohort [median (interquartile range), 7.6 (6.3 to 9.8) pmol · L^−1^ in women and 12.5 (10.5 to 15.5) pmol · L^−1^ in men] (7).

A study in rats supported a beneficial role of increased water intake and decreased circulating vasopressin for metabolic health; this study demonstrated a favorable effect on metabolism when vasopressin was reduced by increased hydration, whereas sustained vasopressin infusion impaired glucose tolerance (14). These data, together with a Mendelian randomization study, in which genetic variation in the human vasopressin gene was recently associated with both elevated copeptin and increased risk for hyperglycemia in men, but not in women (15), supports causality between elevated copeptin and metabolic disease.

In this study, we found a small but significant reduction in fasting plasma glucose after 6 weeks of water intervention (Fig. 3). The glucose-lowering effect was driven by participants whose copeptin concentration remained high from population screening to pilot baseline investigation ~3 years later [Fig. 4(b)], as well as by participants with the highest baseline copeptin concentrations [Fig. 4(a)], who were also the most pronounced water responders (Table 3). These data complement our previous finding, that water responders express a water-induced reduction of the diabetogenic hormone glucagon (28). The finding that individuals with repeatedly high copeptin concentrations are driving the glucose-lowering effect from increased hydration may be related to a higher probability that these individuals truly have long-term low water intake and a lower probability that they have a temporary elevated copeptin (due to, for example, short-term dehydration, a minor infection, or stress). Because our study lasted 6 weeks, we believe that it is still premature to define exactly which segment of the population, based on copeptin concentration and markers of hydration status, obtains a clinically meaningful metabolic effect by hydration.

Copeptin is elevated in conditions of acute illness. For example, heart failure, acute myocardial infarction, hemorrhage, and sepsis result in marked elevation of copeptin (35–38). In the general population, however, after exclusion of other known factors, the observed elevated copeptin concentration might be due to low total water intake. In the current study, participants in the top tertile of baseline copeptin, with a median (interquartile range) copeptin concentration of 35.2 (21.0 to 66.5) pmol · L^−1^ , did not differ in clinical characteristics from participants with moderately elevated baseline copeptin except for having a greater reduction in copeptin and fasting glucose after increased hydration [Table 3; Fig. 4(a)]. Thus, we have no indication that underlying medical conditions or medication use differ between individuals in the top tertile of copeptin concentration and participants with moderately elevated copeptin. Note that these individuals are selected mainly on the basis of several indices of low water intake, including elevated copeptin concentration, and that these high copeptin concentrations thus may be considered as the upper normal limit in an otherwise healthy population with low water intake.

Regarding safety, water supplementation did not induce any change in plasma osmolality or erythrocyte volume fraction. Plasma sodium was slightly reduced at 6 weeks compared with baseline, but the water intervention did not induce any cases of hyponatremia (defined as plasma sodium < 135 mmol · L^−1^). This was not surprising because overconsumption of water that exceeds the maximal renal excretion rate of 0.7 to 1.0 L per hour is difficult to achieve and because all participants were selected on the basis of several indices of habitual low water intake. In fact, during the 6-week intervention, the participants increased their daily total water intake only enough to reach levels classified as adequate (2.0 to 2.5 L per day) (Table 4) (39).

We conclude that water supplementation in persons with habitual low water intake is effective in lowering copeptin and appears to be a safe and promising intervention that may lower fasting plasma glucose and thus reduce diabetes risk. Further investigations are warranted to assess the long-term effectiveness of water supplementation in lowering blood glucose concentration and, consequently, diabetes risk.

## Abbreviations:

eGFR: estimated glomerular filtration rate
MOS: Malmö Offspring Study
